# Bidirectional associations between irritable bowel syndrome and psychological distress: a longitudinal population-based study

**DOI:** 10.1017/S0033291726103328

**Published:** 2026-02-11

**Authors:** Ya-Ju Yu, Yao-Ching Huang, Tsu-Hsuan Weng, Hsiang-Ying Huang, Fu-Chih Lai, Tzu-Chiao Lin, Pi-Ching Yu, Wu-Chien Chien

**Affiliations:** 1 Graduate Institute of Life Sciences, College of Biomedical Sciences, National Defense Medical University, Taiwan; 2 Department of Chemical Engineering and Biotechnology, National Taipei Institute of Technology: National Taipei University of Technology (Taipei Tech), Taiwan; 3 Department of Medical Research, Tri-Service General Hospital, National Defense Medical University, Taiwan; 4 Master Program in School of Nursing, College of Nursing, Taipei Medical University, Taiwan; 5 Post-Baccalaureate Program in Nursing, College of Nursing, Taipei Medical University, Taiwan; 6 Division of Colorectal Surgery, Department of surgery, Tri-Service General Hospital, Taiwan; 7 Graduate Institute of Medical Sciences, College of Medicine, National Defense Medical Center, Taiwan; 8 Graduate Institute of Public Health, College of Public Health, National Defense Medical University, Taiwan

**Keywords:** anxiety, bidirectional association, cohort study, depression, gut–brain axis, irritable bowel syndrome (IBS), psychological distress, sleep disorders (SDs)

## Abstract

**Background:**

Irritable bowel syndrome (IBS) commonly co-occurs with psychological distress, including depression and anxiety, but the temporal and bidirectional nature of this relationship remains unclear. Dysregulation of the gut–brain–microbiota axis has been proposed as a shared mechanism.

**Methods:**

We conducted two retrospective, population-based cohort studies using Taiwan’s National Health Insurance Research Database (2000–2015). Cohort 1 assessed the risk of incident IBS among patients with newly diagnosed depression or anxiety, while Cohort 2 evaluated the risk of subsequent depression or anxiety among patients with newly diagnosed IBS. Propensity score matching, multivariable Cox regression, and Fine–Gray competing risk models were applied.

**Results:**

IBS was associated with increased risks of depression (adjusted hazard ratio [aHR] = 1.55) and anxiety (aHR = 1.68). Conversely, depression and anxiety were associated with higher risks of developing IBS (aHR = 1.45 and 1.51, respectively). Associations were stronger among females and younger adults aged 18–39 years. Sleep disorders (SDs) showed the strongest modifying effect in both directions (sub-distribution HR ≈ 1.60). Results were consistent across sensitivity analyses.

**Conclusions:**

This nationwide longitudinal study demonstrates a robust bidirectional association between IBS and psychological distress, supporting integrated screening and multidisciplinary care approaches targeting gut–brain interactions.

## Introduction

Irritable bowel syndrome (IBS) is a chronic disorder of gut–brain interaction characterized by recurrent abdominal pain and altered bowel habits in the absence of identifiable structural abnormalities. Globally, IBS affects ~10–15% of adults and imposes a substantial burden on quality of life, work productivity, and healthcare utilization (Canavan, West, & Card, 2014; Lovell & Ford, 2012). Although IBS is not life-threatening, its chronic and unpredictable nature contributes to long-term psychosocial stress and functional impairment comparable to other chronic medical conditions.

Psychological distress – including depression and anxiety – is highly prevalent among individuals with IBS. Meta-analytic evidence suggests that 40–60% of IBS patients meet criteria for an anxiety disorder and up to 30–50% meet criteria for depressive disorders, substantially exceeding rates in the general population (Fond et al., 2014; Lee et al., 2017; Zamani, Alizadeh-Tabari, & Zamani, 2019). This comorbidity has shifted IBS from being viewed solely as a gastrointestinal disorder to a multifactorial biopsychosocial condition that involves interactions among neural, immune, endocrine, and microbial systems (Carabotti, Scirocco, Maselli, & Severi, 2015; Ford, Lacy, & Talley, 2020; Holtmann, Ford, & Talley, 2022).

A central conceptual framework linking IBS with psychological distress is the gut–brain–microbiome axis, a bidirectional communication pathway integrating neuroendocrine stress responses, autonomic function, immune activity, and microbial metabolites (Cryan et al., 2019; Mayer, Tillisch, & Gupta, 2015; O’Mahony et al., 2015). Dysregulation along this axis – including altered serotonin signaling, increased intestinal permeability, mast-cell activation, low-grade inflammation, and microbial dysbiosis – has been implicated in both IBS and affective disorders (Carabotti et al., 2015; Foster & McVey Neufeld, 2013; Valles-Colomer et al., 2019). Chronic stress and mood disorders may heighten visceral hypersensitivity via hypothalamic–pituitary–adrenal (HPA) axis dysregulation, while IBS-related pain and bowel symptoms can induce emotional distress and hypervigilance, creating a self-perpetuating symptom cycle.

Recent genetic research further strengthens the biological plausibility of this bidirectional link. Large-scale genome-wide association studies (GWAS) from the UK Biobank and international consortia have reported significant genetic correlations between IBS and major depressive disorder, generalized anxiety disorder, and neuroticism, suggesting partially shared polygenic architecture (Saito et al., 2023; Visscher et al., 2017; Xu et al., 2024). Several IBS-associated loci map to genes involved in serotonergic signaling, epithelial barrier integrity, immune and inflammatory pathways, and neural pain processing – biological domains also implicated in mood and anxiety disorders. These convergent findings indicate that IBS and psychological distress may arise from overlapping genetic vulnerabilities rather than entirely independent disease processes.

Prior epidemiological studies also demonstrate age- and sex-specific differences in these associations. Women not only have a higher prevalence of IBS overall but also show stronger comorbidity with both depression and anxiety than men, with pooled estimates indicating higher odds of mood and anxiety disorders among female IBS patients (Lee et al., 2017; Videlock, Mayer, & Chang, 2023; Zamani et al., 2019). In terms of age, several population-based and longitudinal studies suggest that younger adults, typically those in early to mid-adulthood (~18–39 years), exhibit more pronounced associations between IBS and stress-related or affective disorders compared with middle-aged and older adults, possibly reflecting heightened psychosocial stress, developmental vulnerability, and lifestyle factors (Wester et al., 2023). These patterns highlight the importance of examining potential age- and sex-specific susceptibility when evaluating IBS-psychological distress relationships.

Despite extensive research, most prior studies have been cross-sectional or based on clinical samples, limiting causal inference and generalizability. Only a few longitudinal, population-based studies have examined temporal ordering, and even fewer have explicitly addressed the bidirectional nature of the IBS-psychological distress relationship with rigorous methods and competing-risk models (Kim et al., 2022; Wang et al., 2023; Zhu et al., 2022). Furthermore, the magnitude of risk across demographic subgroups and comorbidity profiles remains insufficiently characterized.

To address these gaps, we conducted two complementary bidirectional cohort analyses using Taiwan’s National Health Insurance Research Database (NHIRD), a large and nationally representative dataset with long-term follow-up. Our objectives were: (1) to examine whether individuals with depression or anxiety have an elevated risk of developing IBS, and (2) to determine whether individuals with IBS are at increased risk of subsequent depression or anxiety. We hypothesized that significant bidirectional associations would emerge and that these associations would be stronger among women, younger adults (18–39 years), and individuals with specific comorbidities such as sleep disorders (SDs) and peptic ulcer disease. Clarifying these temporal and subgroup-specific relationships is essential for informing integrated clinical care and advancing mechanistic understanding of gut–brain interactions.

## Methods

### Data source

This bidirectional cohort study was conducted using data from the Taiwan NHIRD, which covers more than 99% of Taiwan’s population and provides longitudinal claims data for outpatient visits, hospital admissions, diagnostic codes (ICD-9-CM), prescriptions, and demographic characteristics. The NHIRD has been validated for epidemiologic research, demonstrating high accuracy for major diagnoses (Cheng et al., 2014; Hsieh et al., 2019).

To improve diagnostic accuracy and reduce misclassification, we utilized the Longitudinal Health Insurance Database (LHID), a representative subset of one million randomly sampled beneficiaries followed between 2000 and 2015. Individuals with incomplete demographic information (i.e. missing age, sex, or insured premium) were excluded. Participants younger than 18 years were also excluded according to the study design. A detailed description of sampling procedures, representativeness, and attrition is provided in Supplementary Table S1.

Of the initial one million individuals in the LHID, 1,982 individuals (0.20%) had incomplete demographic information (missing age, sex, urbanization level, or insured income category) and were excluded. An additional 2,614 individuals (0.26%) were younger than 18 years and removed according to the study design. These exclusions resulted in a fully analyzable adult cohort with complete demographic and enrollment data before applying matching procedures.

### Study design and population

Two complementary retrospective cohort analyses were conducted to examine the bidirectional associations between psychological distress (depression and anxiety) and IBS.

#### Cohort 1: Psychological distress predicting IBS

We identified individuals newly diagnosed with depressive or anxiety disorders between January 1, 2002, and December 31, 2012. The index date was defined as the first qualifying psychiatric diagnosis. To enhance diagnostic validity, inclusion required ≥2 outpatient visits or ≥1 inpatient hospitalization with a relevant diagnosis within 12 months.

**Depressive disorders** included the following ICD-9-CM–defined conditions:

- **Major depressive disorder (MDD):** 296.2 and 296.3
- **Dysthymic disorder:** 300.4
- **Depressive disorder, not otherwise specified (NOS):** 311

**Anxiety disorders** included:

- **Generalized anxiety disorder (GAD):** 300.02
- **Panic disorder:** 300.01
- **Phobic disorders:** 300.20–300.29
- **Acute stress reaction/acute stress disorder:** 308.3
- **Post-traumatic stress disorder (PTSD):** 309.81
- **Adjustment disorder with anxiety features:** 309.24
- **Anxiety state, unspecified:** 300.00

To enhance diagnostic validity, cases were required to have **≥2 outpatient visits or ≥1 inpatient diagnosis** within a 12-month period.

Individuals with a prior diagnosis of IBS (ICD-9-CM: 564.1) before the index date were excluded to ensure incident outcome assessment. Each case was matched 1:1 with a control without psychological distress based on age, sex, and index year using propensity score matching.

Participants were followed until incident IBS, withdrawal from insurance, death, or December 31, 2015.

#### Cohort 2: IBS predicting psychological distress

In the reverse analysis, individuals newly diagnosed with IBS between 2002 and 2012 were identified using the same diagnostic criteria (≥2 outpatient visits or ≥1 inpatient claim). Those with any depressive or anxiety disorder before the index date were excluded to ensure incident outcome assessment. IBS cases were matched 1:1 with controls without IBS using the same matching procedure.

Participants were followed until new-onset depression or anxiety, withdrawal, death, or the study endpoint.

**Figure 1.**
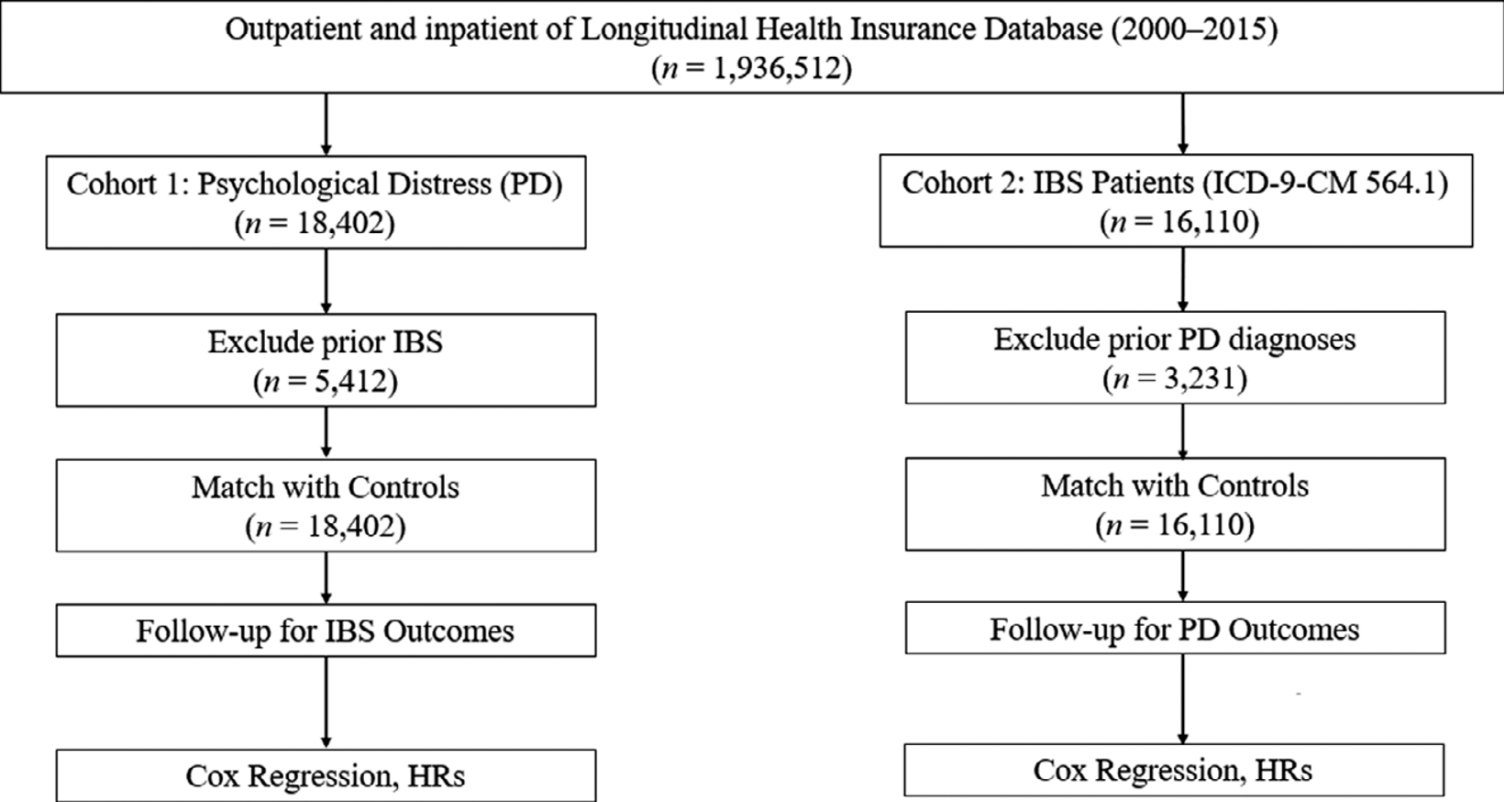
Sample selection flowchart.

A consolidated flowchart including all exclusions (prior IBS, prior psychological distress, and missing data) and final analytic sample sizes is shown in Figure 1.

**Cohort 1: Psychological distress → Subsequent IBS**

- Initial LHID sample: **1,000,000**
- Missing demographic information: **1,982 excluded**
- Age < 18 years: **2,614 excluded**
- Prior IBS diagnosis (ICD-9-CM 564.1): **5,412 excluded**
- Eligible population for PD cohort: **990,0xx**
- Newly diagnosed depressive/anxiety disorders (2002–2012): **18,402**
- After 1:1 matching with controls:
- Psychological distress group: **18,402**
- Matched controls: **18,402**
- **Final follow-up *N* for IBS outcome**: **36,804**

**Cohort 2: IBS → Subsequent psychological distress**

- Newly diagnosed IBS cases (2002–2012): **16,110**
- Excluded prior depressive or anxiety disorders: **3,231 excluded**
- After matching 1:1 with controls:
- IBS cohort: **16,110**
- Matched controls: **16,110**
- **Final** follow-up ***N* for PD outcomes**: **32,220**

### Covariates

Covariates were selected a priori based on existing literature and clinical plausibility rather than solely on data-driven criteria. We adjusted for age, sex, urbanization level, and monthly income because these sociodemographic factors are well-established determinants of both IBS and affective disorders in population-based studies and reflect differences in healthcare access, lifestyle, and socioeconomic status. Urbanization level was classified using the National Health Research Institutes’ four-level urbanization index (Level 1 = most urbanized; Level 4 = rural), and monthly income was derived from insured payroll-related premiums and grouped into three categories (<NT$18,000, NT$18,000–34,999, and ≥ NT$35,000).

We additionally included hypertension, diabetes mellitus, hyperlipidemia, coronary artery disease (CAD), chronic obstructive pulmonary disease (COPD), peptic ulcer disease, and SDs as medical covariates. These conditions were chosen because they (1) are common in adults, (2) have been linked to both IBS and depression/anxiety through shared pathways, such as chronic low-grade inflammation, HPA axis dysregulation, autonomic and vascular changes, and illness-related stress, and (3) are among the most reliably coded diagnoses in the NHIRD. Each comorbidity was defined using ICD-9-CM/ICD-10-CM codes recorded within 1 year before the index date.

We did not include every available diagnosis in the database, such as rare conditions or highly overlapping cardiometabolic diagnoses, to avoid overadjustment and multicollinearity, which may reduce model stability and obscure the interpretation of the main exposure–outcome associations. Thus, the final covariate set was intended to capture the major sociodemographic and cardiometabolic comorbidity burden relevant to both IBS and psychological distress, while maintaining parsimony and robustness of the regression models. (Gracie, Hamlin, & Ford, 2013; Wang et al., 2022).

#### Demographics

- **Age** (continuous; categorized: 18–39, 40–64, and ≥65 years)
- **Sex**
- **Urbanization level**: defined according to population density, educational attainment, proportion of elderly residents, and availability of medical resources (levels 1–4; 1 = most urban)
- **Monthly income**: defined using National Health Insurance premium brackets (≤NT$17,280; NT$17,281–22,800; ≥NT$22,801)

**Comorbidities** (ICD-9-CM codes verified within 1 year before index date):

- Hypertension
- Diabetes mellitus
- Hyperlipidemia
- CAD
- COPD
- Peptic ulcer disease
- SDs (insomnia, sleep apnea, and parasomnias)

#### Healthcare utilization

- Number of outpatient visits in the prior year (proxy for illness severity and healthcare-seeking behavior)

### Statistical analysis

All statistical analyses were conducted separately for the two cohorts: (1) psychological distress predicting incident IBS and (2) IBS predicting incident psychological distress. Baseline characteristics were summarized using means and standard deviations for continuous variables and counts and percentages for categorical variables. Group differences were examined using *t*-tests or chi-square tests as appropriate.

To estimate the association between exposure and outcomes, we used Cox proportional hazards regression models, reporting adjusted hazard ratios (aHRs) with 95% confidence intervals (CIs). Time to event was calculated from the index date to the diagnosis of the outcome, withdrawal from the National Health Insurance program, death, or the end of follow-up, whichever occurred first. All models were adjusted for sociodemographic and clinical covariates selected a priori based on prior literature and biological plausibility, including age, sex, urbanization level, monthly income, hypertension, diabetes mellitus, hyperlipidemia, CAD, COPD, peptic ulcer disease, and SDs. Proportional hazards assumptions were examined using Schoenfeld residuals, with no meaningful violations detected.

#### Interaction analyses

To formally assess whether the association between exposure and outcome differed by sex or age, we included multiplicative interaction terms in the Cox models. Specifically, sex × exposure and age group × exposure terms were added to the fully adjusted models. Age was modeled as a categorical variable with four levels (18–24, 25–44, 45–64, and ≥65 years). Statistical significance of interaction terms was evaluated using likelihood ratio tests comparing models with and without the interaction term. Significant interaction terms indicate that the exposure–outcome association differs across sex or age categories.

#### Sensitivity analyses

Several sensitivity analyses were performed to evaluate the robustness of the findings:

1. excluding individuals diagnosed with the outcome within the first year after the index date to reduce reverse causation.
2. restricting analyses to participants with at least two outpatient diagnoses or one inpatient diagnosis to enhance diagnostic validity.
3. conducting stratified analyses by sex and age group. These analyses were consistent with the primary results.

All analyses were performed using SAS version 9.4 (SAS Institute, Cary, NC, USA). All statistical tests were two-sided, with an alpha level of 0.05 considered statistically significant. Because the primary hypotheses were prespecified and focused on two directional associations (psychological distress → IBS and IBS → psychological distress), no formal adjustment for multiple comparisons was applied to these primary analyses. Secondary analyses, including stratified analyses and interaction tests, were interpreted with caution, and results were presented alongside 95% CIs to allow readers to evaluate effect size and precision.

### Ethical considerations

This study was approved by the Institutional Review Board of Tri-Service General Hospital (TSGHIRB: E202516037). Written informed consent was waived due to the use of anonymized secondary data. NHIRD data security and encryption procedures follow Taiwan’s national regulations (Ministry of Health and Welfare, 2023).

## Results

### Baseline characteristics: Cohort 1 (Psychological distress predicting IBS)

Table 1 presents the baseline characteristics of individuals with psychological distress and their matched controls. A total of 468,722 participants were included, with 234,361 individuals in each group after matching.

**Table 1.** Baseline characteristics of study participants: Cohort 1 (Psychological distress predicting IBS)

Variable	With psychological distress (*n* = 234,361)	Without psychological distress (*n* = 234,361)	*p*-value
**IBS, *n* (%)**			<0.001
Without IBS	159,117 (67.89%)	206,151 (87.96%)	
With IBS	75,244 (32.11%)	28,210 (12.04%)	
**Gender, *n* (%)**			1.000
Male	123,710 (52.79%)	123,710 (52.79%)	
Female	110,651 (47.21%)	110,651 (47.21%)	
**Age (years), mean ± SD**	42.96 ± 15.00	42.96 ± 15.00	1.000
**Age group, *n* (%)**			1.000
18–24	27,397 (11.69%)	27,397 (11.69%)	
25–44	103,948 (44.35%)	103,948 (44.35%)	
45–64	81,284 (34.68%)	81,284 (34.68%)	
≥65	21,732 (9.27%)	21,732 (9.27%)	
**Urbanization level, *n* (%)**			1.000
Level 1 (most urbanized)	72,652 (31.0%)	72,652 (31.0%)	
Level 2	89,057 (38.0%)	89,057 (38.0%)	
Level 3	37,498 (16.0%)	37,498 (16.0%)	
Level 4 (rural)	35,154 (15.0%)	35,154 (15.0%)	
**Monthly income (NT$), *n* (%)**			<0.001
<18,000	68,706 (29.32%)	123,273 (52.60%)	
18,000–34,999	106,477 (45.43%)	76,994 (32.85%)	
≥35,000	59,178 (25.25%)	34,094 (14.55%)	
**Comorbidities, *n* (%)**			<0.001
COPD	120,045 (51.22%)	54,787 (23.38%)	
CAD	79,922 (34.10%)	28,413 (12.12%)	
Hyperlipidemia	121,897 (52.01%)	59,879 (25.55%)	
Diabetes mellitus	74,081 (31.61%)	39,593 (16.89%)	
Hypertension	121,233 (51.73%)	70,926 (30.26%)	

*Note:* Values are presented as number (percentage) or mean ± standard deviation (SD). *p*-values were calculated using Chi-square or Fisher’s exact test for categorical variables and *t*-tests for continuous variables. Urbanization level was categorized according to the National Health Research Institutes’ 4-level urbanization index (Level 1 = most urbanized; Level 4 = least urbanized). Monthly income was based on insured payroll-related premiums (<NT$18,000, NT$18,000–34,999, and ≥NT$35,000).Comorbidities were defined using ICD-9-CM/ICD-10-CM codes within 1 year before the index date. IBS, irritable bowel syndrome; COPD, chronic obstructive pulmonary disease; CAD, coronary artery disease.

#### Sociodemographic characteristics

The two groups were well balanced with respect to age (mean 42.96 years, SD = 15.00) and **sex** distribution (52.79% male and 47.21% female). The urbanization level was identical across groups due to the matching design, with:

- 31.0% residing in Level 1 (most urbanized) areas
- 38.0% in Level 2
- 16.0% in Level 3
- 15.0% in Level 4 (rural regions)

Monthly income differed significantly between groups (*p* < .001). Participants with psychological distress were more likely to belong to higher income categories (45.43% vs. 32.85% in the NT$18,000–34,999 level; 25.25% vs. 14.55% in the ≥NT$35,000 level).

#### Medical comorbidities

Across all comorbidity categories, individuals with psychological distress had substantially higher prevalence of chronic conditions, including:

- COPD (51.22% vs. 23.38%)
- CAD (34.10% vs. 12.12%)
- Hyperlipidemia (52.01% vs. 25.55%)
- Diabetes mellitus (31.61% vs. 16.89%)
- Hypertension (51.73% vs. 30.26%)

All differences were statistically significant (*p* < .001).

#### IBS prevalence

The prevalence of IBS at baseline was significantly higher in the psychological distress group compared to controls (32.11% vs. 12.04%, *p* < .001), indicating a strong association between psychological distress and IBS in the raw data before multivariable analysis.

### Baseline characteristics: Cohort 2 (IBS predicting psychological distress)

Table 2 summarizes the baseline characteristics of 361,258 participants, divided evenly into IBS (*n* = 180,629) and non-IBS controls (*n* = 180,629).

**Table 2.** Baseline characteristics of study participants: Cohort 2 (IBS predicting psychological distress)

Variable	IBS(*n* = 180,629)	Without IBS(*n* = 180,629)	*p*-value
**Psychological distress, *n* (%)**			<0.001
Without PD	97,948 (54.23%)	148,971 (82.47%)	
With PD	82,681 (45.77%)	31,658 (17.53%)	
**Type of distress**			<0.001
Depression	15,806 (19.12%)	7,339 (23.18%)	
Anxiety	66,875 (80.88%)	24,319 (76.82%)	
**Gender, *n* (%)**			1.000
Male	91,509 (50.66%)	91,509 (50.66%)	
Female	89,120 (49.34%)	89,120 (49.34%)	
**Age (years), mean ± SD**	46.66 ± 16.35	46.66 ± 16.35	1.000
**Age group, *n* (%)**			1.000
18–24	17,211 (9.53%)	17,211 (9.53%)	
25–44	67,837 (37.56%)	67,837 (37.56%)	
45–64	66,504 (36.82%)	66,504 (36.82%)	
≥65	29,077 (16.10%)	29,077 (16.10%)	
**Urbanization level, *n* (%)**			1.000
Level 1 (most urbanized)	55,995 (31.0%)	55,995 (31.0%)	
Level 2	68,639 (38.0%)	68,639 (38.0%)	
Level 3	28,900 (16.0%)	28,900 (16.0%)	
Level 4 (rural)	27,095 (15.0%)	27,095 (15.0%)	
**Monthly income (NT$), *n* (%)**			<0.001
<18,000	43,194 (23.91%)	99,860 (55.28%)	
18,000–34,999	85,106 (47.12%)	55,902 (30.95%)	
≥35,000	52,329 (28.97%)	24,867 (13.77%)	
**Comorbidities, *n* (%)**			<0.001
COPD	99,184 (42.32%)	43,793 (18.69%)	
CAD	61,766 (26.36%)	25,080 (10.70%)	
Hyperlipidemia	98,960 (42.23%)	48,627 (20.75%)	
Diabetes mellitus	61,926 (26.42%)	32,278 (13.77%)	
Hypertension	96,745 (41.28%)	60,629 (25.87%)	

*Note:* Values are presented as number (percentage) or mean ± standard deviation (SD). *p*-values were calculated using Chi-square or Fisher’s exact test for categorical variables and *t*-tests for continuous variables. Urbanization level was categorized according to the National Health Research Institutes’ 4-level index (Level 1 = highly urbanized; Level 4 = rural). Monthly income reflects insured premium levels used in the National Health Insurance system. Psychological distress includes depressive and anxiety disorders. Comorbidities were identified using ICD-9-CM/ICD-10-CM codes.PD, psychological distress; COPD, chronic obstructive pulmonary disease; CAD, coronary artery disease.

#### Psychological distress **outcomes**

The prevalence of psychological distress was substantially higher among individuals with IBS (45.77%) compared with controls (17.53%, *p* < .001). Among those with psychological distress, anxiety disorders were more common than depressive disorders in both groups.

#### Sociodemographic characteristics

The IBS and control groups were well matched on **age** (mean 46.66 years, SD = 16.35), **sex** (50.66% male; 49.34% female), and age group distribution.

Urbanization level also showed an identical distribution due to matching:

- Level 1: 31.0%
- Level 2: 38.0%
- Level 3: 16.0%
- Level 4: 15.0%

#### Monthly income

Income distribution differed significantly (*p* < .001):

- The IBS group had a higher proportion in middle income (47.12% vs. 30.95%)
- Non-IBS controls had a higher proportion in the lowest-income category (55.28% vs. 23.91%)

#### Medical comorbidities

Participants with IBS exhibited consistently higher rates of medical comorbidities, including:

- COPD (42.32% vs. 18.69%)
- CAD (26.36% vs. 10.70%)
- Hyperlipidemia (42.23% vs. 20.75%)
- Diabetes mellitus (26.42% vs. 13.77%)
- Hypertension (41.28% vs. 25.87%)

All differences were statistically significant (*p* < .001).

### Cohort 1: Depression or anxiety predicting incident IBS (Psychological distress → IBS)

After adjusting for age, sex, comorbidities, and healthcare utilization, patients with depression or anxiety had a 55% increased risk of developing IBS compared to controls (aHR = 1.55, 95% CI: 1.42–1.69, *p* < 0.001; Table 3). Subgroup analyses showed that the risk was higher among females (aHR = 1.68, 95% CI: 1.50–1.88) than males (aHR = 1.39, 95% CI: 1.21–1.59), and greatest among younger adults aged 18–39 years (aHR = 1.74, 95% CI: 1.52–1.99). The association was also more pronounced among individuals with comorbidities such as SDs (aHR = 1.62, 95% CI: 1.45–1.81), peptic ulcer disease (aHR = 1.49, 95% CI: 1.33–1.67), and hyperlipidemia (aHR = 1.41, 95% CI: 1.25–1.59). Significant associations were also observed in patients with hypertension, diabetes mellitus, COPD, and CAD, indicating that baseline medical complexity may amplify IBS risk in patients with psychological distress.

**Table 3.** Risk of developing IBS following psychological distress (Cox regression analysis): Cohort 1 (Psychological distress → IBS)

Subgroup	aHR (95% CI)	*p*-value
**Overall**	1.55 (1.42–1.69)	<0.001
**Sex**		
Female	1.68 (1.50–1.88)	<0.001
Male	1.39 (1.21–1.59)	<0.001
**Age group (years)**		
18–39	1.74 (1.52–1.99)	<0.001
40–64	1.48 (1.31–1.67)	<0.001
≥65	1.32 (1.10–1.58)	<0.001
**Comorbidities**		
Hypertension	1.43 (1.28–1.59)	<0.001
Diabetes mellitus (DM)	1.38 (1.20–1.58)	<0.001
COPD	1.35 (1.12–1.63)	0.001
Coronary artery disease	1.31 (1.15–1.49)	<0.001
Hyperlipidemia	1.41 (1.25–1.59)	<0.001
Peptic ulcer	1.49 (1.33–1.67)	<0.001
Sleep disorders	1.62 (1.45–1.81)	<0.001

*Note:* Adjusted hazard ratios (aHRs) were derived from multivariable Cox proportional hazards regression models. Models were adjusted for age, sex, income, urbanization, healthcare utilization, and baseline comorbidities. Subgroup analyses assess whether the association between psychological distress and incident IBS varies by sex, age group, and presence of specific comorbidities. CI, confidence interval; aHR, adjusted hazard ratio; COPD, chronic obstructive pulmonary disease; DM, diabetes mellitus; CAD, coronary artery disease.

During a mean follow-up period of 7.3 years, 1,498 individuals (8.1%) in the psychological distress group developed IBS, compared to 921 (5.0%) in the control group. Kaplan–Meier survival analysis demonstrated a significantly higher cumulative incidence of IBS in the exposed group (log-rank test, *p* < 0.001; Supplementary Figure S1).

### Cohort 2: IBS predicting incident depression or anxiety

After adjusting for age, sex, comorbidities, and healthcare utilization, patients with IBS had a 46% increased risk of developing psychological distress (depression or anxiety) compared to matched controls (aHR = 1.46, 95% CI: 1.37–1.56, *p* < 0.001; Table 4). Subgroup analyses indicated that this association was stronger in females (aHR = 1.52, 95% CI: 1.39–1.66) than in males (aHR = 1.38, 95% CI: 1.24–1.54), and most evident in individuals aged 18–39 years (aHR = 1.58, 95% CI: 1.40–1.78). Among patients with comorbidities, the risk of psychological distress was highest in those with SDs (aHR = 1.60, 95% CI: 1.45–1.76), peptic ulcer disease (aHR = 1.44, 95% CI: 1.30–1.59), and hyperlipidemia (aHR = 1.35, 95% CI: 1.21–1.50). Consistent elevations in risk were also observed in those with hypertension, diabetes, COPD, and CAD, underscoring the potential interaction between gastrointestinal and systemic conditions in the development of psychological comorbidities.

**Table 4.** Risk of developing depression or anxiety following IBS: Cohort 2 (IBS predicting psychological distress)

Subgroup	aHR (95% CI)	*p*-value
**Overall**	1.46 (1.37–1.56)	<0.001
**Sex**		
Female	1.52 (1.39–1.66)	<0.001
Male	1.38 (1.24–1.54)	<0.001
**Age group (years)**		
18–39	1.58 (1.40–1.78)	<0.001
40–64	1.42 (1.28–1.57)	<0.001
≥65	1.29 (1.12–1.49)	<0.001
**Comorbidities**		
Hypertension	1.41 (1.28–1.55)	<0.001
Diabetes mellitus (DM)	1.36 (1.21–1.53)	<0.001
COPD	1.32 (1.13–1.54)	<0.001
Coronary artery disease	1.28 (1.14–1.43)	<0.001
Hyperlipidemia	1.35 (1.21–1.50)	<0.001
Peptic ulcer	1.44 (1.30–1.59)	<0.001
Sleep disorders	1.60 (1.45–1.76)	<0.001

*Note:* Adjusted hazard ratios (aHRs) were derived from Cox proportional hazards models. Models were adjusted for age, sex, income, urbanization level, outpatient visits, and comorbidities. CI, confidence interval; aHR, adjusted hazard ratio; COPD, chronic obstructive pulmonary disease; DM, diabetes mellitus; CAD, coronary artery disease.

In the reverse analysis, during a mean follow-up of 6.9 years, 2,031 IBS patients (12.6%) developed either depression or anxiety, compared to 1,308 (8.1%) in the control group. The cumulative incidence curves showed a statistically significant divergence over time (log-rank *p* < 0.001; Supplementary Figure S2).

### Sensitivity and subgroup analyses

#### Subgroup analysis in competing risk models (Cohort 1: Psychological distress predicting IBS)

In competing risk models adjusting for death as a competing event, the associations between common comorbidities and incident IBS remained robust. Among individuals with SDs, the sub-distribution hazard ratio (sub-HR) for developing IBS was 1.67 (95% CI: 1.40–1.95), indicating a significantly increased risk compared to those without SDs. Similarly, elevated risks were observed for other comorbidities such as COPD (sub-HR = 1.55, 95% CI: 1.30–1.85), diabetes mellitus (sub-HR = 1.40, 95% CI: 1.22–1.60), and peptic ulcer (sub-HR = 1.42, 95% CI: 1.19–1.70) (Supplementary Figure S3).

#### Subgroup analysis in competing risk models (Cohort 2: IBS predicting psychological distress)

In competing risk models adjusting for death as a competing event, the associations between IBS and the development of psychological distress (depression or anxiety) remained consistent across all examined subgroups. The sub-HRs demonstrated statistically significant elevations in risk across demographic and comorbidity-based strata.

Among patients with SDs, the sub-HR for developing psychological distress was 1.60 (95% CI: 1.45–1.76), indicating a markedly increased vulnerability compared to those without sleep disturbances. Similarly, IBS patients with peptic ulcer disease also showed elevated risk (sub-HR = 1.44; 95% CI: 1.30–1.59). Additional comorbidities such as hyperlipidemia (sub-HR = 1.35; 95% CI: 1.21–1.50), CAD (sub-HR = 1.28; 95% CI: 1.14–1.43), and COPD (sub-HR = 1.32; 95% CI: 1.13–1.54) were also associated with significantly increased risk of psychological outcomes following IBS diagnosis. Stratified by sex and age, the risk was notably higher in females (sub-HR = 1.52; 95% CI: 1.39–1.66) and in younger adults aged 18–39 years (sub-HR = 1.58; 95% CI: 1.40–1.78) (Supplementary Figure S4).

### Bidirectional associations between IBS and psychological distress by subgroup

Supplementary Figure S5 shows the bidirectional associations between IBS and depression/anxiety by subgroup. In competing risk models adjusting for death as a competing event, the bidirectional associations between IBS and psychological distress (depression or anxiety) remained consistent across all examined subgroups. The sub-HRs revealed statistically significant elevations in both directions of risk across demographic and comorbidity-defined strata.

#### Psychological distress → IBS

Among patients with preexisting psychological distress, those with SDs had the highest risk of developing IBS (sub-HR = 1.62; 95% CI: 1.45–1.81), followed by those with peptic ulcer disease (sub-HR = 1.49; 95% CI: 1.33–1.67) and hyperlipidemia (sub-HR = 1.41; 95% CI: 1.25–1.59). Other comorbid conditions, such as hypertension (sub-HR = 1.43) and DM (sub-HR = 1.38), also showed elevated IBS risk.

Stratified analyses revealed a stronger association in females (sub-HR = 1.68; 95% CI: 1.50–1.88) and in younger individuals aged 18–39 years (sub-HR = 1.74; 95% CI: 1.52–1.99), suggesting age- and sex-related susceptibility.

#### IBS → Psychological distress

Conversely, patients with IBS demonstrated significantly increased risks of subsequent psychological distress across all subgroups. Those with comorbid SDs exhibited the greatest vulnerability (sub-HR = 1.60; 95% CI: 1.45–1.76), followed by peptic ulcer disease (sub-HR = 1.44; 95% CI: 1.30–1.59) and hyperlipidemia (sub-HR = 1.35; 95% CI: 1.21–1.50).

Elevated risks were also observed in patients with CAD (sub-HR = 1.28) and COPD (sub-HR = 1.32). The female subgroup (sub-HR = 1.52; 95% CI: 1.39–1.66) and those aged 18–39 years (sub-HR = 1.58; 95% CI: 1.40–1.78) again showed stronger associations, mirroring the pattern seen in the reverse analysis.

### Interaction analyses

To evaluate whether the associations differed across demographic strata, we conducted multiplicative interaction analyses.

The sex × exposure interaction was statistically significant in both cohorts (Psychological distress → IBS: *p* < 0.001; IBS → Psychological distress: *p* < 0.001), indicating that the strength of association differed between men and women.

Similarly, the age group × exposure interaction was significant in both directions (Psychological distress → IBS: *p* < 0.001; IBS → Psychological distress: *p* < 0.01).

Specifically, the associations were consistently stronger among females compared with males and were most pronounced among younger adults aged 18–39 years, in both directions of the IBS–psychological distress relationship.

Full model estimates for all interaction terms are provided in Supplementary Tables S2 and S3.

## Discussion

This large, population-based cohort study provides robust evidence for a bidirectional association between IBS and psychological distress, including depression and anxiety. Using two complementary longitudinal cohorts, Fine–Gray competing risk models, and extensive subgroup analyses, we demonstrate that individuals with psychological distress exhibit a significantly increased risk of later developing IBS, and patients with IBS similarly show elevated risk for subsequent depression and anxiety. These findings reinforce the conceptualization of IBS as a disorder of gut–brain interaction involving interconnected neural, immunological, microbial, and psychological pathways.

### Comparison with existing literature

The current results align with prior research documenting high rates of depression and anxiety among IBS populations (Fond et al., 2014; Lee et al., 2017). However, most previous studies have been cross-sectional or conducted in clinical samples, limiting temporal inference. Our study extends prior evidence by simultaneously demonstrating bidirectional temporal relationships in a single population-based dataset with long-term follow-up.

These findings are consistent with earlier longitudinal work showing that mood disorders increase the risk of later IBS (Kim et al., 2022) and that IBS predicts later psychiatric disorders (Zhu et al., 2022). Wang et al. (2023) reported a similar bidirectional association between anxiety and IBS in a 10-year cohort. The present study strengthens the literature by providing national-level estimates with rigorous matching procedures, competing-risk adjustments, and detailed stratification by sex and age.

### Biological mechanisms and GWAS evidence

A growing literature implicates the gut–brain–microbiome axis in the shared pathophysiology underlying IBS and psychological distress. Chronic stress and affective disorders may heighten visceral sensitivity via dysregulation of the HPA axis, alterations in serotonin signaling, and immune-mediated pathways (Chrousos, 2022; O’Mahony et al., 2015). Conversely, IBS-related dysbiosis may influence mood through microbial metabolites – including short-chain fatty acids and tryptophan derivatives – that affect neuroinflammation, neurotransmission, and emotional regulation (Foster & McVey Neufeld, 2013; Valles-Colomer et al., 2019).

Recent genomic research offers additional support for shared etiological mechanisms. Large-scale GWAS have documented significant genetic correlations between IBS, major depressive disorder, generalized anxiety disorder, and neuroticism (Saito et al., 2023; Xu et al., 2024). Overlapping loci include genes involved in serotonergic signaling, epithelial barrier function, inflammatory responses, and neural pain circuits-biological domains relevant to both IBS symptom generation and mood dysregulation (Visscher et al., 2017). These convergent findings suggest that IBS and psychological distress share polygenic vulnerability rather than reflecting independent disease processes.

### Age- and sex-related differences

Our findings of stronger bidirectional associations among women and younger adults are consistent with prior literature. The more pronounced associations observed in women and younger adults suggest age- and sex-related vulnerability along gut–brain pathways, potentially reflecting hormonal modulation, stress responsivity, and developmental factors (Wester et al., 2023). Women have a higher prevalence of IBS and report greater symptom severity, pain sensitivity, and stress responsivity, potentially due to hormonal modulation of serotonergic and visceral pain pathways (Videlock et al., 2023). Younger adults (18–39 years) also exhibited higher risks in both directions, a pattern observed in other longitudinal studies (Wester et al., 2023). This vulnerability may reflect heightened exposure to psychosocial stressors, early-life adversity, lifestyle patterns, or developmental neurobiological factors.

SDs emerged as the strongest modifier of risk in both directions. This finding is biologically plausible because circadian disruption can alter gut motility, inflammatory tone, neuroendocrine signaling, and microbial rhythmicity – all of which may exacerbate mood dysregulation and gastrointestinal sensitivity (Benedict et al., 2020). The consistent influence of peptic ulcer disease, metabolic conditions, and COPD suggests broader systemic interplay among inflammation, stress physiology, and gut–brain pathways.

### Clinical implications

These findings underscore the need for integrated, multidisciplinary approaches in managing IBS and psychological distress. Given the bidirectional risk, clinicians should implement routine screening: gastroenterologists should assess depressive and anxiety symptoms, and mental health providers should inquire about gastrointestinal symptoms.

Psychological treatments – including cognitive-behavioral therapy, gut-directed hypnotherapy, and mindfulness-based interventions – have demonstrated sustained benefits for both IBS and psychiatric symptoms (Laird et al., 2022; Pinto-Sanchez et al., 2022). Pharmacological treatments, such as low-dose tricyclic antidepressants and selective serotonin reuptake inhibitors, also target shared mechanisms involving pain modulation and emotional regulation (Black, Drossman, Talley, & Ford, 2022). Emerging interventions, including probiotics, prebiotics, and microbiome-targeted therapies, further highlight the potential of gut–brain modulation in improving outcomes (Distrutti, Monaldi, Ricci, & Fiorucci, 2016).

### Public health and research implications

From a public health perspective, early detection of comorbid symptoms may reduce chronic disability and healthcare burden. Younger adults, women, and individuals with SDs or metabolic comorbidities may benefit from targeted screening programs or digital mental health tools. Lifestyle interventions addressing sleep hygiene, stress management, and diet may also play preventive roles.

Future research should integrate multi-omics approaches – including genomics, metabolomics, and microbiome profiling – to identify mechanistic subtypes of IBS and psychiatric comorbidity (Rodiño-Janeiro et al., 2023). Longitudinal brain imaging and microbiome studies may clarify the temporal interplay between neural circuits, microbial states, and symptoms. Randomized controlled trials should evaluate gut–brain-targeted interventions using biomarkers to advance precision medicine for these disorders.

### Strengths and limitations

Major strengths include the nationally representative cohort, the bidirectional design, rigorous propensity score matching, competing risk analyses, and long-term follow-up. The large sample enabled robust subgroup and interaction analyses. However, limitations include reliance on administrative diagnostic codes, absence of IBS subtype or symptom severity data, lack of lifestyle factors (diet, substance use, and trauma exposure), and the absence of biological measures, such as inflammatory markers or microbiome profiles. Our findings apply only to incident cases, as individuals with any history of IBS or prior depressive/anxiety disorders were excluded from both cohorts. Therefore, the temporal associations reported here may not generalize to patients with preexisting conditions, whose symptom trajectories and etiologic pathways may differ. The Taiwanese population context may also limit generalizability to non-Asian populations.

## Conclusion

This nationwide, longitudinal cohort study provides robust epidemiological evidence supporting a bidirectional association between IBS and psychological distress, specifically depression and anxiety. By applying competing risk models and conducting comprehensive stratified analyses, we demonstrate that individuals with IBS are at significantly increased risk of developing mood disorders, and conversely, those with preexisting depression or anxiety are more likely to develop IBS. These associations are particularly pronounced among younger adults (18–39 years), females, and individuals with comorbid conditions such as SDs, peptic ulcer disease, and cardiometabolic diseases.

Our findings reinforce the conceptualization of IBS as a multisystem, biopsychosocial disorder involving complex interactions along the gut–brain–microbiota axis. Clinically, these results highlight the need for integrated screening and multidisciplinary management strategies across gastroenterology and mental health care. From a public health perspective, early identification and intervention in high-risk groups may mitigate the chronic disease burden and improve patient outcomes. Future research should incorporate multi-omics data, longitudinal microbiome and neuroimaging assessments, and randomized controlled trials of gut–brain-targeted therapies to advance precision medicine approaches for these highly comorbid conditions.

## Supporting information

10.1017/S0033291726103328.sm001 Yu et al. supplementary material Yu et al. supplementary material

## Data Availability

The data that support the findings of this study are available from the corresponding author upon reasonable request.

## Supplementary material

The supplementary material for this article can be found at http://doi.org/10.1017/S0033291726103328.
