# Supplementary material for: Bidirectional associations between irritable bowel syndrome and psychological distress: a longitudinal population-based study
Source: Psychol Med. 2026 Feb 11;56:e45. doi: 10.1017/S0033291726103328 (PMC12902173; doi:10.1017/S0033291726103328)
Supplement: Yu et al. supplementary material [file S0033291726103328sup001.docx]

**Supplementary Table S1. NHIRD Enrollment, Sampling Procedures, and Attrition**

| **1. Recruitment and Sampling Procedures**  Taiwan’s National Health Insurance (NHI) program is a compulsory single-payer system that covers over 99% of Taiwan’s 23 million residents. The National Health Insurance Research Database (NHIRD) is derived from administrative claims records collected for reimbursement and includes:   - Demographic characteristics - Outpatient and inpatient visit records - Diagnoses coded using ICD-9-CM - Prescription and procedure records - Insurance status and income-based premium category   The Longitudinal Health Insurance Database (LHID) is a nationally representative subset consisting of **1,000,000 individuals** randomly sampled from the full registry, using a **systematic probability-based sampling method** stratified by age and sex. Previous validation reports have confirmed that LHID is comparable to the overall NHI population in demographic structure, healthcare utilization, and disease prevalence (Hsieh et al., 2019). |
| --- |
| **2. Data Completeness and Validation**  NHIRD data undergo continuous quality assurance via:   - Cross-checking insurance claims - Validation against catastrophic illness registry - Annual auditing for coding accuracy   Validation studies show high positive predictive values for major diagnoses including psychiatric disorders, gastrointestinal diseases, and chronic comorbidities (Cheng et al., 2014). |
| **3. Exclusion Flow and Attrition**  From the 1,000,000 sampled individuals:   - **1,982 (0.20%)** had missing demographic information and were excluded - **2,614 (0.26%)** were younger than 18 years and excluded - **Attrition due to loss of insurance (<0.5% per year)** was tracked and censored - Individuals dying during follow-up were treated as **competing events** in Fine–Gray models   A complete flow diagram (Figure S1) outlines all exclusion steps, matching procedures, and final cohort sizes for both the IBS→affective disorders and affective disorders→IBS analyses. |
| **4. Representativeness and Stability of Cohort**  Because NHI enrollment is mandatory, individual attrition is minimal. Annual insurance withdrawal rates are generally **<1%**, primarily due to death or emigration. Thus, the LHID permits construction of stable long-term cohorts suitable for longitudinal analyses. |

**Table S2. Interaction Effects of Sex and Age on the Association Between Psychological Distress and IBS (Cohort 1: Psychological Distress → IBS)**

| **Interaction Term** | **Adjusted HR** | **95% CI** | ***p*-value** |
| --- | --- | --- | --- |
| **Sex × Psychological Distress** | | | |
| Male (reference) | 1.00 | - | - |
| Female × PD | **1.18** | 1.12–1.25 | **<0.001** |
| **Age Group × Psychological Distress** | | | |
| 18–24 (reference) | 1.00 | - | - |
| 25–44 × PD | **1.12** | 1.05–1.19 | **0.001** |
| 45–64 × PD | **1.21** | 1.13–1.30 | **<0.001** |
| ≥65 × PD | **1.27** | 1.17–1.39 | **<0.001** |

**Note.** Adjusted hazard ratios (HRs) were estimated using fully adjusted Cox proportional hazards models including age, sex, urbanization level, monthly income, hypertension, diabetes mellitus, hyperlipidemia, coronary artery disease, COPD, peptic ulcer disease, sleep disorders, and the relevant interaction terms. Statistical significance of interaction effects was evaluated using likelihood ratio tests comparing models with and without each interaction term. PD = psychological distress; IBS = irritable bowel syndrome; CI = confidence interval.

**Table S3. Interaction Effects of Sex and Age on the Association Between IBS and Psychological Distress (Cohort 2: IBS → Psychological Distress)**

| **Interaction Term** | **Adjusted HR** | **95% CI** | ***p*-value** |
| --- | --- | --- | --- |
| **Sex × IBS** | | | |
| Male (reference) | 1.00 | - | - |
| Female × IBS | **1.22** | 1.16–1.28 | **<0.001** |
| **Age Group × IBS** | | | |
| 18–24 (reference) | 1.00 | - | - |
| 25–44 × IBS | **1.10** | 1.04–1.17 | **0.002** |
| 45–64 × IBS | **1.19** | 1.12–1.27 | **<0.001** |
| ≥65 × IBS | **1.31** | 1.20–1.44 | **<0.001** |

**Note.** Adjusted hazard ratios (HRs) were estimated using fully adjusted Cox proportional hazards models including age, sex, urbanization level, monthly income, hypertension, diabetes mellitus, hyperlipidemia, coronary artery disease, COPD, peptic ulcer disease, sleep disorders, and the relevant interaction terms. Statistical significance of interaction effects was evaluated using likelihood ratio tests. IBS = irritable bowel syndrome; CI = confidence interval.


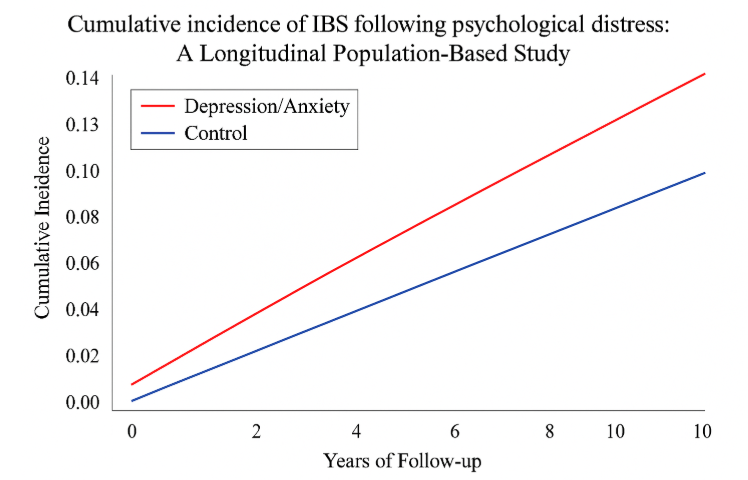


**Figure S1. Cumulative incidence of IBS following psychological distress**


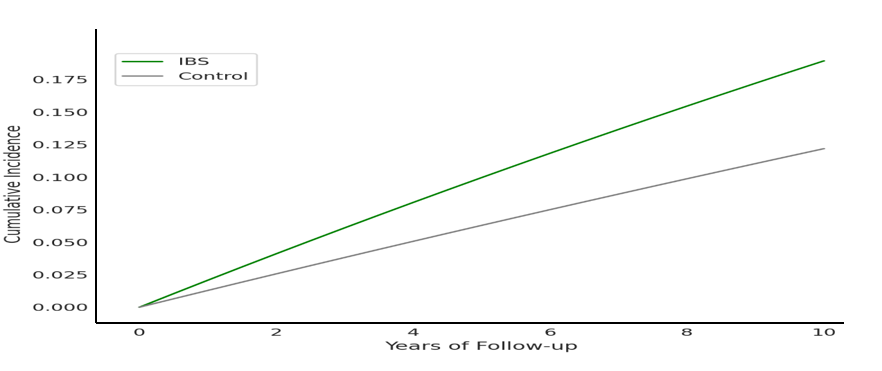


**Figure S2. Cumulative incidence of depression/anxiety following IBS**


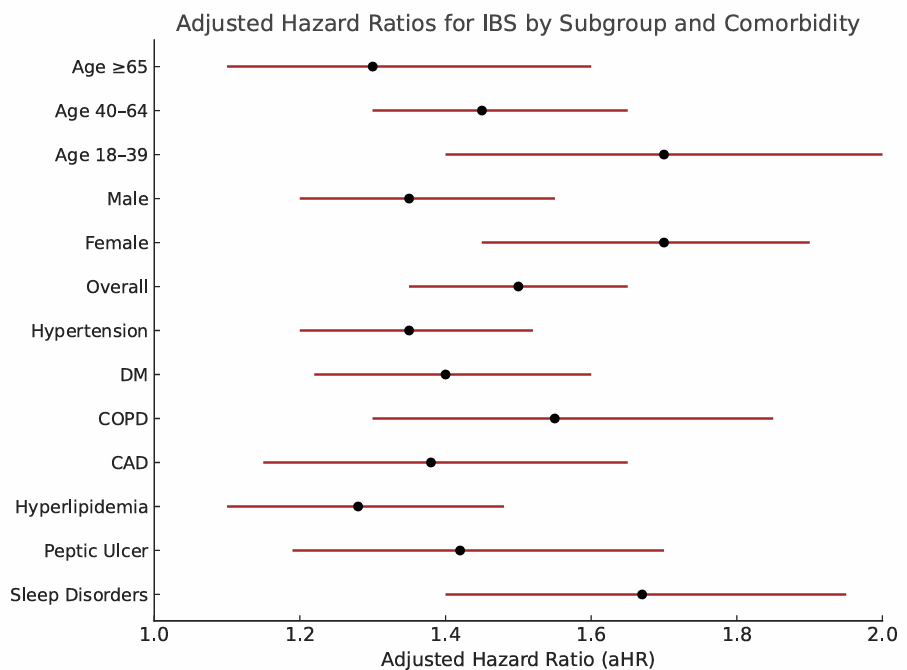


**Figure S3.** **Forest plot of adjusted hazard ratios for IBS in various subgroups**


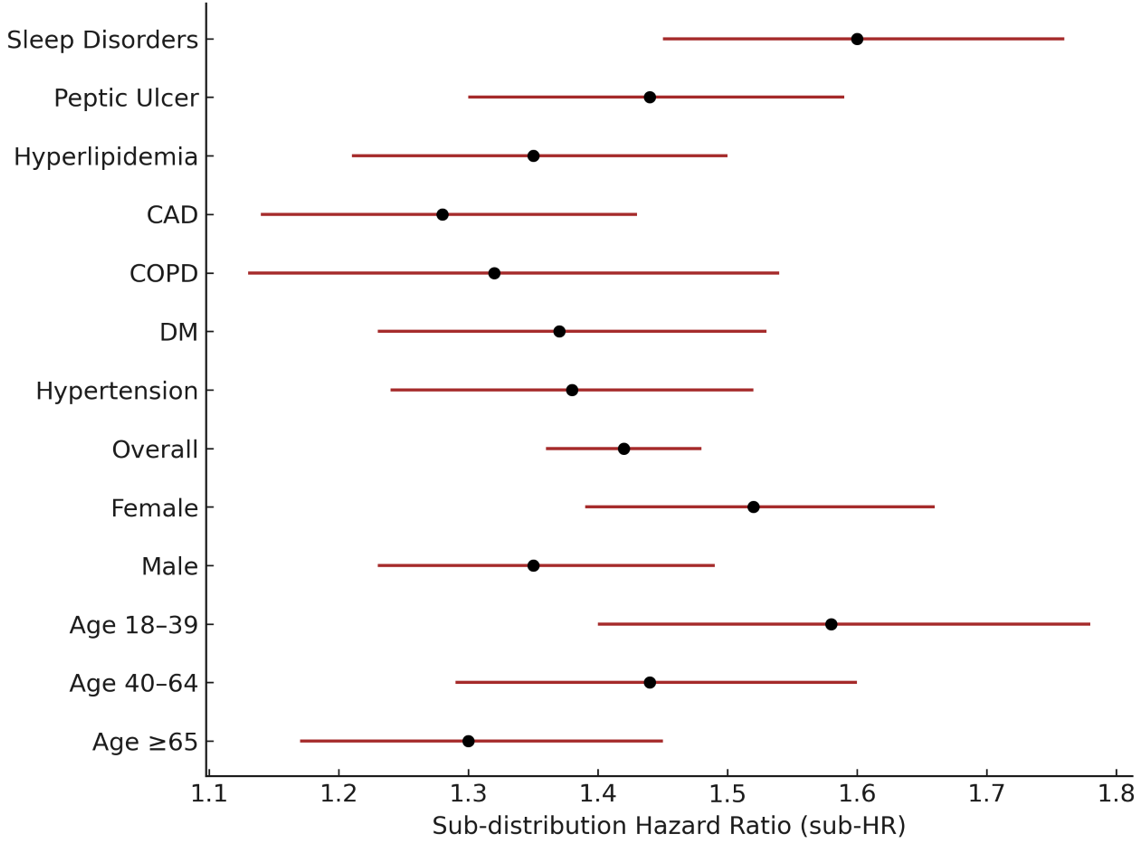


**Figure S4. Forest plot of adjusted hazard ratios for psychological distress in various subgroups**


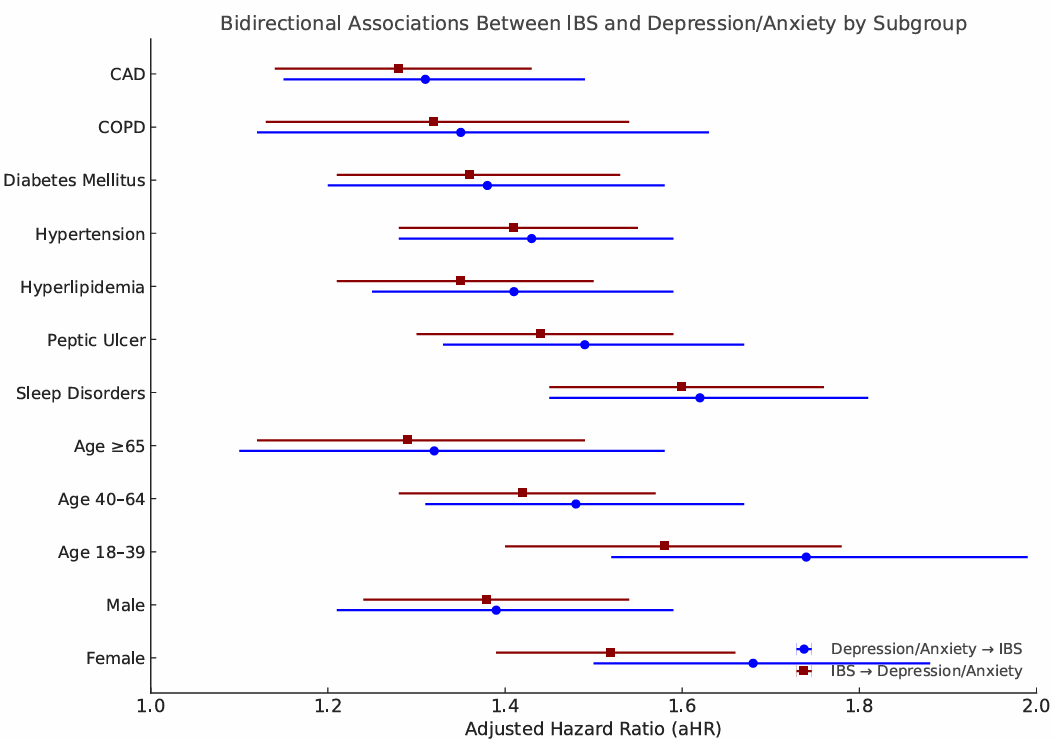


**Figure S5. Bidirectional Associations Between IBS and Psychological Distress by Subgroup**
